# COVID-19 vaccine hesitancy and its associated factors in Malaysia

**DOI:** 10.1371/journal.pone.0266925

**Published:** 2022-09-01

**Authors:** Kai Wei Lee, Lai Ti Gew, Ching Sin Siau, Suat Cheng Peh, Yook Chin Chia, Shakila Yacob, Nee Nee Chan, Vei Ken Seow, Pei Boon Ooi

**Affiliations:** 1 Faculty of Medicine and Health Sciences, Department of Pre-Clinical Sciences, Universiti Tunku Abdul Rahman, Kajang, Malaysia; 2 Centre for Research on Communicable Diseases, Universiti Tunku Abdul Rahman, Kajang, Malaysia; 3 Department of Biological Sciences, School of Medical & Life Sciences, Sunway University, Bandar Sunway, Selangor, Malaysia; 4 Faculty of Health Sciences, Centre for Community Health Studies (ReaCH), Universiti Kebangsaan Malaysia, Kuala Lumpur, Malaysia; 5 Jeffrey Sachs Center on Sustainable Development, Sunway University, Bandar Sunway, Selangor, Malaysia; 6 Department of Medical Sciences, School of Medical & Life Sciences, Sunway University, Bandar Sunway, Selangor, Malaysia; 7 Faculty of Medicine, Department of Primary Care Medicine, University of Malaya, Kuala Lumpur, Malaysia; 8 Faculty of Arts & Social Sciences, Department of History, University of Malaya, Kuala Lumpur, Malaysia; 9 Faculty of Social Sciences, UCSI University Kuala Lumpur, Kuala Lumpur, Malaysia; 10 Emergency Medicine Department, Sunway Medical Centre, Bandar Sunway, Selangor, Malaysia; 11 Liveable Cities, Future Cities Research Institute, Sunway University, Bandar Sunway, Selangor, Malaysia; Johns Hopkins Bloomberg School of Public Health, UNITED STATES

## Abstract

The success of the COVID-19 vaccination programme to achieve herd immunity depends on the proportion of the population inoculated. COVID-19 vaccination hesitancy is a barrier to reaching a sufficient number of people to achieve herd immunity. This study aims to determine the prevalence of COVID-19 vaccine hesitancy and to identify the reasons contributing to vaccine hesitancy using the Theory of Planned Behavior. A cross-sectional online survey was conducted between May 2021 to June 2021. Using exponential non-discriminative snowball sampling, participants were recruited via social media and telecommunication platforms. We used a questionnaire that obtained information on participant socio-demographics, vaccine hesitancy, pseudoscientific practices, conspiracy beliefs, subjective norms, perceived behavioural control, main reasons for not intending to get the COVID-19 vaccine; influential leaders, gatekeepers and anti-or pro-vaccination lobbies; and global vaccine hesitancy. A total of 354 responses (mean age = 32.5 years old ±13.6; 70.3% females) were included for analysis. The prevalence of COVID-19 vaccine hesitancy was 11.6%. COVID-19 vaccine hesitancy was significantly and positively associated with those who agreed with influential leaders, gatekeepers, and anti- or pro-vaccination lobbies (adjusted B coefficient = 1.355, *p* = 0.014), having a “wait and see” attitude to see if the COVID-19 vaccine is safe (adjusted B coefficient = 0. 822, *p* <0.001), perceiving that the vaccine will give them COVID-19 (adjusted B coefficient = 0.660, *p* <0.002), planned to use masks/others precautions instead (adjusted B coefficient = 0.345, *p* = 0.038) and having higher scores in conspiracy beliefs (adjusted B coefficient = 0.128, *p* <0.001). Concern about the costs associated with the vaccine (adjusted B coefficient = -0.518, *p* <0.001), subjective norms (adjusted B coefficient = -0.341, *p* <0.001), and perceived behavioural control (adjusted B coefficient = -0.202, p = 0.004) were negatively associated with vaccine hesitancy. COVID-19 vaccine hesitancy in Malaysia is low. Several factors were identified as being associated with vaccine hesitancy. Factors associated with vaccine hesitancy would be useful in tailoring specific interventions involving positive messages by influential leaders, which address vaccine misinformation and the wait-and-see attitude which may delay the uptake of COVID-19 vaccines.

## Introduction

As of 10 August 2021, there were 203,295,170 cases of COVID-19 globally whilst in Malaysia, there was a total of 1,299,767 cases and 11,162 deaths [1]. Several measures were put into place to counter the disease transmission including social distancing, wearing a mask, and frequent sanitization [2]. However, some of these measures hindered daily activities and were not fully adopted by many; the daily positive cases also continued to rise. The limited ability to comply with these preventive measures to curb the COVID-19 pandemic has thus made vaccines essential in helping to control disease transmission.

There is strong evidence that COVID-19 vaccines can reduce the odds of disease severity and mortality [3], yet many people are still hesitant, and this may delay vaccine uptake [4]. According to MacDonald et al. [5], vaccine hesitancy is defined as a delay or refusal of the vaccine. Vaccine hesitancy is a complex issue. It is context and time-specific, such that the underlying reasons differ from place to place and across vaccine types. It is also influenced by behavioural and attitudinal factors, such as complacency, convenience, and confidence [5]. Based on the 2013 WHO-UNICEF Joint Reporting Forms (JRF) immunization forum, the top three reasons for vaccine hesitancy were beliefs, attitudes, and motivations regarding health and prevention [6]. The risk-benefit opinion on vaccines includes perceived risks from information shared through public media and social communication, such as vaccine side effects and anti-vaccination reports, as well as direct and indirect personal experiences [7]. Thus, the objective of this study is to elucidate which factors are most closely associated with vaccine hesitancy among Malaysians.

**Fig 1:**
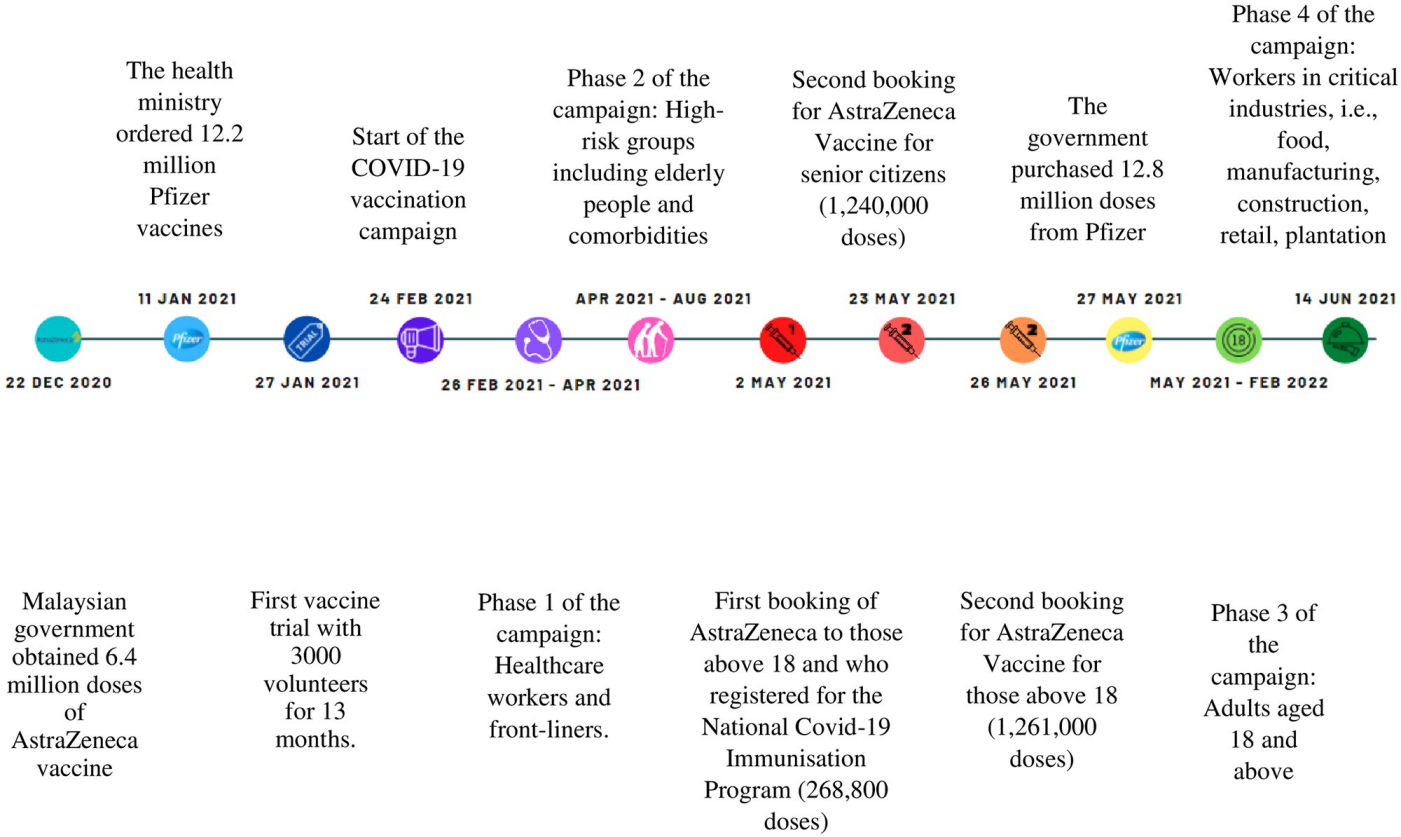
Malaysia’s timeline on COVID-19 vaccination initiatives and activities.

The official roll-out date of the mass COVID-19 vaccination programme in Malaysia was 24 February 2021. The first phase was vaccination of the front liners, followed by the second phase, whereby priority was given to high-risk groups, such as senior citizens (≥60 years old), people with disabilities, and those with underlying chronic health conditions. At the early stages of the vaccination programme, negative perceptions of the vaccine were widespread. In particular, the arrival of the first batch of the Oxford-AstraZeneca vaccine on 23 April 2021 was not welcomed as there was a concern then due to widespread reports on the side effect of blood clotting. However, when it was subsequently made known that the occurrence of the blood clotting side effect was no more common than the usual numbers as seen before the COVID-19 pandemic, the fear was alleviated and the vaccine was then offered to the Malaysian public on a voluntary “first come, first served” basis. The timeline is illustrated below (Fig 1).

Wong et al. surveyed Malaysians in April 2020, a month after the first nationwide lockdown was implemented and before the vaccination programme had started [8]. They reported that 94.3% of the 1159 participants indicated a positive intention to receive a vaccine. However, in another study by Bono et al. between December 2020 and February 2021, across nine Low- and Middle-Income Countries in which Malaysia was included, the result showed that the vaccine acceptance rate was only 55.4% of the participants surveyed if the vaccine was 90% effective [9]. Hence, we aimed to examine vaccine hesitancy and identify the factors associated with COVID-19 vaccine hesitancy. This study thus hopes to provide valuable insights into the reasons surrounding the people’s hesitancy, what their concerns are, and what influenced their decision to vaccination.

In this study, we used the Theory of Planned Behaviour (TPB) to examine the factors associated with COVID-19 vaccine hesitancy. The TPB model has been widely used in explaining how the health behaviour of people could change after a considerable amount of information is made known to them [10,11]. According to TPB, any logical and reasoned health decision is guided based on three considerations: a) behavioural beliefs (an individual’s belief in the positive outcomes of their behaviour), b) normative beliefs (an individual’s society and familial pressure to perform the behaviour), and c) control beliefs (an individual’s ability and control to perform the behaviour) [12]. Combinations of all three considerations predicted a person’s intention to perform the wanted health behaviour [10]. As an expectancy-value model, in the past, TPB has been widely used in health research to explain the expectations, motivations or beliefs of individuals before a decision is made [13,14]. Recently, the TPB model has been adopted in studies examining the intention in getting the COVID-19 vaccination [15–17], where the individuals’ intention was higher when they perceived the COVID-19 vaccination to be beneficial to them and believed in their doctor’s advice in getting the COVID-19 vaccine. Additionally, a study by Hossain et al. [18] found that the TPB model showed a higher predictability level of vaccine hesitancy compared to the Health Belief Model and the other psychological antecedents, such as confidence, constraints, complacency, calculation, and collective responsibility. Besides that, it is known that an individual’s close relations can influence the individual’s intent, whereby individuals with close friends and family who approve of the vaccine appear to have a stronger intention to be vaccinated [19]. Hence, subjective norms (i.e., perceived social pressure to comply or not in getting the COVID-19 vaccination) were added to the model.

**Fig 2:**
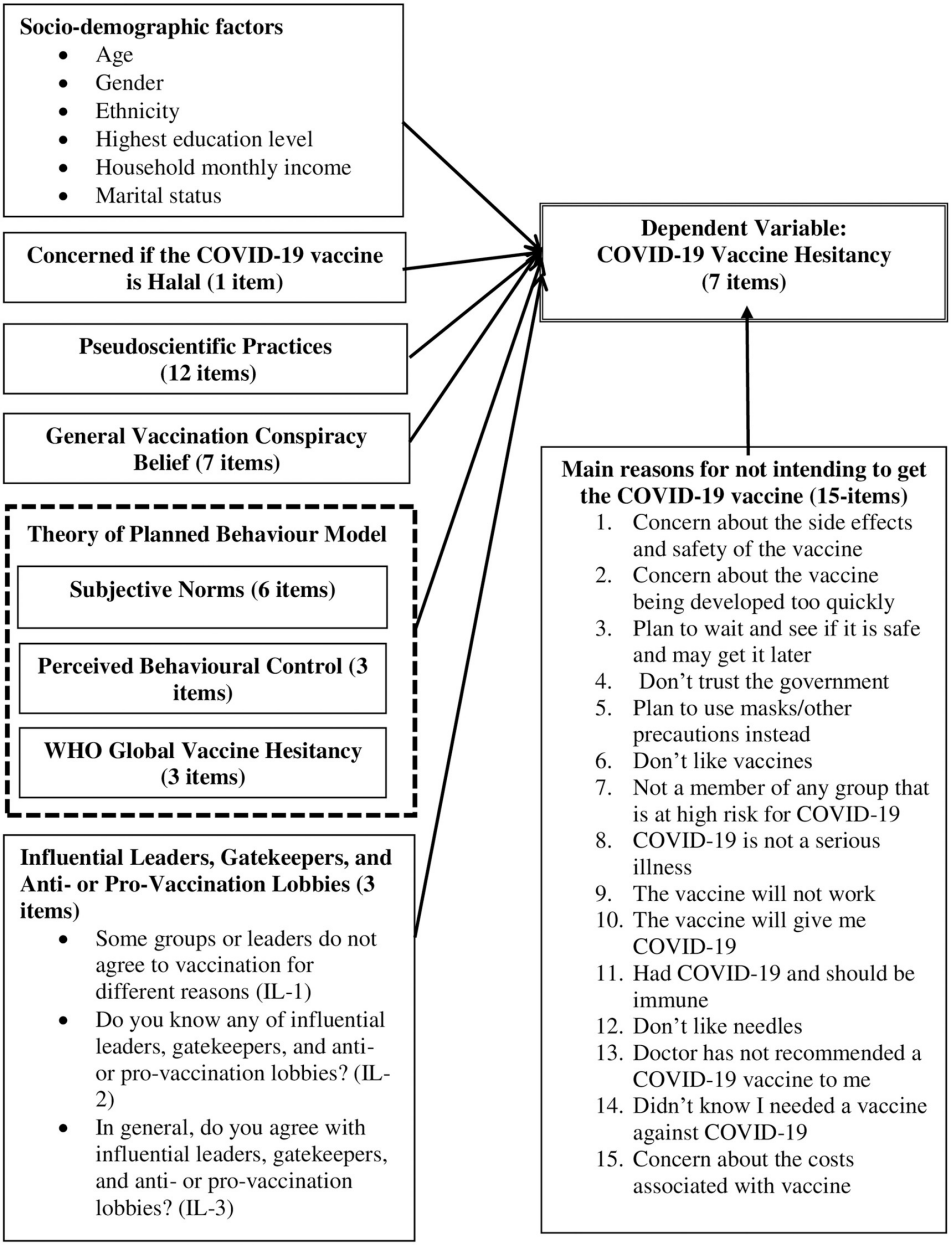
Conceptual framework of the study: The associated factors and vaccine hesitancy. Note: 1. Dotted lines: Factors that are parts of the Theory of Planned Behaviour. 2. Solid lines: Independent variables which are included as parts of the extended Theory of Planned Behaviour. 3. Double lines: Dependent variable.

As a public health crisis, the COVID-19 pandemic has brought about many conspiracy theories. Those who believe in COVID-19 conspiracy theories tend not to want to follow or adhere to COVID-19 guidelines and are less likely to get vaccinated when a vaccine becomes available [20]. Studies have also shown that those believing in pseudoscientific practices were more likely to support claims about the efficacy of alternative and complementary medical treatments. Conspiracy theories may influence an individual by increasing distrust, making them less inclined to heed the recommendation. Thus, we have also included the pseudoscientific practices [20] and vaccine conspiracy beliefs [21] in our study. Additionally, global vaccine hesitancy, as well as reasons for hesitancy were also included in the framework of this study, as these components have been reported to influence vaccine hesitancy [22,23]. For example, a narrative review found that being against vaccines, in general, was associated with higher hesitancy toward COVID-19 vaccines in particular [22]. In New Zealand, general vaccine hesitancy was also found to be associated with COVID-19 vaccination intention [23]. Here, we present the framework for conceptualizing the association between the expanded TPB model and COVID-19 vaccine hesitancy. This framework is illustrated below (Fig 2).

Based on the literature review, we thus hypothesized, using a multiple factors regression analysis, that the examined factors could be significantly associated with the Oxford COVID-19 vaccine hesitancy. We hope that by expanding the TPB model, a comprehensive association of the various factors that affect the COVID-19 vaccine hesitancy can be identified. Therefore, the objective of this study was to determine the prevalence of COVID-19 vaccine hesitancy and examine the reasons contributing to such a hesitancy.

## Materials and methods

### Study design, population, and sampling methods

The study was a cross-sectional study conducted between 22 May 2021 to 21 June 2021 after the institutional review board approvals were obtained (Sunway University; SUREC 2021/042 and Sunway Medical Centre; 007/2021/IND/ER). Participants were recruited via social media and telecommunication platforms such as Facebook, WhatsApp, and Instagram. Using the exponential non-discriminative snowball sampling method, participants were also encouraged to share the link to the recruitment poster with their network of friends. The inclusion criteria for this study were Malaysians above 18 years old and not yet vaccinated for COVID-19.

### Sample size calculation

The calculated sample size needed for this study was 312 participants at minimum, with a 95% confidence level (z-score of 1.96), and a margin of allowable error set at d = 0.05, given that the estimated prevalence of hesitancy among the general population was 28.4 [24]. By considering the presence of any missing data that will be removed from the analysis, the sample size was increased by 25%, totalling 417 participants. The sample size was calculated using the following formula [25].


$$\text{Sample}\;\text{size}=\frac{Z^{2}\;P\;\left(1-P\right)}{d^{2}}$$

### Study instruments and scoring method

This survey consisted of nine sections.

In Section 1, socio-demographic information was collected (age, gender, ethnicity, highest education level, household monthly income, and marital status). As Malaysia is a predominantly Muslim country, we also asked about the level of concern regarding the Halal status of COVID-19 vaccines, which was adopted from Wong et al, [8] [“I am concerned if the new COVID-19 vaccine is Halal”]. Wong listed only two options, i.e., “Agree” or “Disagree”, which we then modified into a 5-point Likert scale: “Strongly not concerned (scored as 1)”, “Not concerned (scored as 2)”, “Neutral (scored as 3)”, “Concerned (scored as 4)” and “Strongly concerned (scored as 5)”.

In Section 2, we used the Oxford COVID-19 Vaccine Hesitancy Scale, which is a 7-item scale adapted from Freeman et al. [26], for assessing COVID-19 vaccine hesitancy. Item-specific response options, coded from 1 (“Definitely”) to 5 (“Definitely not”) were used. A “Don’t know” option was also provided but was excluded from the scoring. The Cronbach’s alpha for the past study was 0.97. The scale is also proven to be associated with the Vaccine Hesitancy Scale (r = 0.47, p<0.001) [26]. The total score could thus range from 0 to 35, where a higher score would indicate a higher level of vaccine hesitancy. The scores of the 7 items were summed up to give an aggregate score for statistical analysis. The Oxford COVID-19 Vaccine Hesitancy Scale was used to calculate the prevalence of COVID-19 vaccine hesitancy instead of the WHO Global Vaccine Hesitancy as the latter is a generic questionnaire assessing general acceptance/hesitancy toward any vaccines and not specifically for COVID-19 vaccine hesitancy.

Whereas the “Oxford COVID-19 Vaccine Hesitancy-7 items” was designed to estimate provisional willingness to receive a COVID-19 vaccine, it includes beliefs, tightly bound to the willingness to take the vaccine that is plausible drivers of vaccine uptake and closer to our study objective.

In Section 3, the Pseudoscientific Practices Scale was used, which is a 12-item scale adapted from Teovanovic et al. [20]. Participants rated how often they used the pseudoscientific practices in the previous two weeks as a method to protect themselves against or prevent a COVID-19 infection; the rating was done on a scale ranging from 1 (“Never”) to 5 (“Very Often”). The total score was then calculated by summing up the scores of the 12 items. The score could range from 12 to 60; a higher score indicated a higher frequency of pseudoscientific practices used. The summed up scores of the 12 items would give an aggregate score for statistical analysis.

In Section 4, the General Vaccination Conspiracy Beliefs Scale was adapted from Shapiro et al., 2016 [21], with 7 items rated on a 7-points Likert scale, which was converted into a 5-points scale ranging from “Strongly disagree” (scored as 1) to “Strongly agree” (scored as 5). This scale has high internal consistency, with a Cronbach’s alpha of 0.937; it was also validated and showed a moderate relationship with the Conspiracy Mentality Questionnaire in the past study (r = 0.44, p<0.001) [21]. The total score of the scale was calculated by summing up the individual scores of the 7 items. The total score could range from 7 to 35; a higher score indicated a greater endorsement of vaccine conspiracy statements. The summed up scores of the 7 items would give an aggregate score for statistical analysis.

In Section 5, the Subjective Norms Scale, which has 6 items and was originally developed by Ajzen et al. [27], was adapted from Ryan et al. [19], who focused on the antenatal whooping cough vaccine. We rephrased “whooping cough vaccine” to “COVID-19 vaccine”. The Cronbach’s alpha for the past study was 0.82 [19]. The 5-point rating scale included the following options: “Not at all” (scored as 1), “A little” (scored as 2), “Somewhat” (scored as 3), “A lot” (scored as 4) and “A great deal” (scored as 5). We also rephrased “female friend” to “best friend”. The total score was calculated by summing up the individual scores of the 6 items. The total score could range from 6 to 30, where a higher score indicated a greater likelihood to be influenced by others to get vaccinated. The scores of the 6 items were summed up to give an aggregate score for statistical analysis.

In Section 6, the Perceived Behavioral Control Scale, having 3 items and also originating from Ajzen et al., [27], was used by Ryan et al. to measure vaccine hesitancy among pregnant women [19]. The past study reported a Cronbach’s alpha value of 0.71 [19]. Items were rated on a 5-point Likert scale, ranging from “Strongly disagree” (scored as 1) to “Strongly agree” (scored as 5). The total score was calculated by summing up the individual scores of the 3 items. The total score could range from 3 to 15; a higher score indicated a higher level of self-empowerment to get the COVID-19 vaccine. The scores of the 3 items were summed up to give an aggregate score for statistical analysis.

In Section 7, the “Influential leaders, gatekeepers and anti-or pro-vaccination lobbies” Scale consisting of 3 items was used [7]. The first item was “Some groups or leaders do not agree to vaccination for different reasons”; the options given were “Strongly disagree” (scored as 1), “Disagree” (scored as 2), “Neutral” (scored as 3), “Agree” (scored as 4), and “Strongly agree” (scored as 5). The second item was “Do you know any influential leaders, gatekeepers, and anti- or pro-vaccination lobbies?”, and the third item was “In general, do you agree or disagree with these groups?”; the options for the latter two items were either “Yes” or “No”. Each score for the 3 items was presented separately for statistical analysis.

In Section 8, the WHO Global Vaccine Hesitancy Scale-3, originally from Rey et al., [28] and later adapted by Detoc et al., [29] was employed. Global vaccine hesitancy in this scale is defined according to the WHO definition. The items were “Have you ever refused a vaccine for yourself or a child because you considered it useless or dangerous?”, “Have you ever postponed a vaccine recommended by a physician?”, and “Have you ever had a vaccine for a child or yourself despite doubts about its efficacy?”. If a participant answered yes to any one of these items, they were considered to be “global vaccine-hesitant”. The scores of the 3 items were summed up to give an aggregate score for statistical analysis.

The final section, Section 9, was included to find out the participants’ “Main reasons for not intending to get the COVID-19 vaccine”. The section consisted of 15 items [23]. Participants were requested to reflect on the extent to which they agreed with each item as being a reason for not intending to get the COVID-19 vaccine. The response options given were “Strongly disagree” (scored as 1), “Disagree” (scored as 2), “Neutral” (scored as 3), “Agree” (scored as 4), and “Strongly agree” (scored as 5). Each score for the 15 items was presented separately for statistical analysis.

### Statistical analysis

We used the statistical software package IBM SPSS Statistics for Windows, Version 21.0 for the data analysis. Before conducting the formal data analysis, we removed identifications from the dataset which had missing data for at least one variable. We performed descriptive analysis (data were presented in n, %) for socio-demographic characteristics of participants and all scales. The independent samples t-test was used to compare the mean scores for independent variables (two groups only) and Oxford COVID-19 vaccine hesitancy (dependant variable) whereas one-way ANOVA was used to compare mean scores for independent variables which have more than two groups with the Oxford COVID-19 vaccine hesitancy (dependant variable) (Fig 2).

The scores for items were summed up to give an aggregate score that would be used for statistical analysis for these variables: Oxford COVID-19 vaccine hesitancy (7 items), pseudoscientific practices (12 items), general vaccination conspiracy belief (7 items), subjective norms (6 items), perceived behavioural control (3 items). Whereas the scores for individual items were presented separately for these variables: influential leaders, gatekeepers and anti-or pro-vaccination lobbies (3 items); and main reasons for not intending to get the COVID-19 vaccine (15 items). A simple linear regression was done to identify continuous independent variables (i.e., age, pseudoscientific practices, general conspiracy belief, subjective norms, perceived behavioural control, concern whether the COVID-19 vaccine is Halal, some groups or leaders do not agree to vaccination for a different reason, and 15 assorted reasons for not intending to get the COVID-19 vaccine) associated with Oxford COVID-19 Vaccine Hesitancy (dependent variable). Any variables with a *p*-value of <0.25 in the bivariate analysis were entered into the multivariable regression model, with the Oxford COVID-19 vaccine hesitancy as the dependent variable used in the entered model, and variables with a *p*-value of <0.05 were considered statistically significant.

## Results

### Characteristics of participants

A total of 433 responses were obtained in the study, whereby 20 of these responses were ineligible as the participants who provided these responses had already been vaccinated; their responses were thus removed from the analysis. Additionally, 59 responses contained at least one incomplete response (e.g., having a missing value); these responses were also removed. After having removed all ineligible responses, only 354 responses were retained for statistical analysis. Using the Post-hoc statistical power calculator for multiple regression (Soper calculator), we achieved a statistical power of more than 0.8 through the sample size of 354 with 23 predictors, with the observed R squared of 0.531, and the probability level at 0.05. Thus, our collected sample size of n = 433 will be more than adequate for the main objective of this study.

Characteristics of the participants are presented in Table 1. The mean age of the participants was 32.5±13.6 years, Females made up 70.3% of the participants, 57.9% were Chinese, and a majority had completed tertiary education. Slightly more than half (53.4%) of the participants’ monthly income was MYR 2000 or more.

**Table 1:** Socio-demographic characteristics of participants (n = 354).

Characteristics	Category or detail	Overall
**Age**	Mean ± SD	32.5 ±13.6
	Minimum—Maximum	18–85
**Gender, n (%)**	Male	105 (29.7)
	Female	249 (70.3)
**Ethnicity, n (%)**	Malay	56 (15.8)
	Chinese	205 (57.9)
	Indian	69 (19.5)
	Others	24 (6.8)
**Highest education level, n (%)**	Secondary school	24 (6.8)
	Diploma/Degree	221 (62.4)
	Postgraduate	109 (30.8)
**Household monthly income, n (%)**	<MYR 2000	165 (46.6)
	MYR 2000—MYR 3000	34 (9.6)
	MYR 3000—MYR 4000	19 (5.4)
	MYR 4000—MYR 5000	29 (8.2)
	>MYR 5000	107 (30.2)
**Marital status, n (%)**	Single	247 (69.8)
	Married	99 (28.0)
	Divorced	4 (1.1)
	Widowed	4 (1.1)

Note: Data are presented either in Mean ± SD, N (%), or range of minimum and maximum.

### Descriptive analysis of all the assessments used in the survey

A summary of the descriptive analysis of all the measures used in this study is provided in Table 2.

**Table 2:** Descriptive analysis of assessment used (n = 354).

**Oxford COVID-19 Vaccine Hesitancy Scale** **(Total score ranged from 0–35)**	**Mean ± SD**	**11.3 ± 4.9**
	Minimum–maximum	7–32
	Did not endorse any clear vaccine hesitancy response [a response rating of 4 (probably not) or 5 (definitely not)] on any of the 7 items of the scale, n (%)	313 (88.4)
	Endorsed at least one item with a clear vaccine hesitancy response [a response rating of 4 (probably not) or 5 (definitely not)], n (%)	41 (11.6)
	Internal consistency reliability, Cronbach’s alpha value	0.918
**Pseudoscientific Practices Scale** **(Total score ranged from 12–60)**	Mean ± SD	24.4 ± 10.1
	Minimum–maximum	12–56
	Endorsed “Never”, “Rarely” or “Sometimes” [a response rating of 1(never), 2 (rarely) or 3 (sometimes)] on any of the 12 items of the scale, n (%)	146 (41.2)
	Endorsed “Often” or “Very Often” [a response rating of 4 (often) or 5 (very often)] on at least one item of the scale, n (%)	208 (58.8)
	Internal consistency reliability, Cronbach’s alpha value	0.909
**General Vaccination Conspiracy Beliefs Scale** **(Total score ranged from 7–35)**	Mean ± SD	16.5 ± 6.9
	Minimum–maximum	7–35
	Did not endorse any item on conspiracy belief [with a response rating of 1 (strongly disagree), 2 (disagree), or 3 (Neutral)], n (%)	218 (61.6)
	Endorsed at least one item on conspiracy belief [with a response rating of 4 (agree) or 5 (strongly agree)], n (%)	136 (38.4)
	Internal consistency reliability, Cronbach’s alpha value	0.919
**Subjective Norms Scale** **(Total score ranged from 6–30)**	Mean ± SD	22.7 ± 5.0
	Minimum–maximum	8–30
	Internal consistency reliability, Cronbach’s alpha value	0.830
**Perceived Behavioral Control Scale** **(Total score ranged from 3–15)**	Mean ± SD	11.9 ± 2.8
	Minimum–maximum	3–15
	At least one item was not rated with a response rating of 4 (agree) or 5 (Strongly agree), n (%)	144 (40.7)
	All items were rated with a response rating of 4 (agree) or 5 (Strongly agree), n (%)	210 (59.3)
	Internal consistency reliability, Cronbach’s alpha value	0.813
**I am concerned if the new COVID-19 vaccine is Halal**	Strongly not concerned (scored as 1)	235 (66.4)
	Not concerned (scored as 2)	23 (6.5)
	Neutral (scored as 3)	44 (12.4)
	Concerned (scored as 4)	18 (5.1)
	Strongly concerned (scored as 5)	34 (9.6)
**Influential Leaders, Gatekeepers And Anti- Or Pro-Vaccination Lobbies Scale**			
**Some groups or leaders do not agree to vaccination for different reasons (IL-1)**	Strongly disagree (scored as 1)	40 (11.3)
	Disagree (scored as 2)	29 (8.2)
	Neutral (scored as 3)	115 (32.5)
	Agree (scored as 4)	106 (29.9)
	Strongly agree (scored as 5)	64 (18.1)
**Do you know of any influential leaders, gatekeepers and anti- or pro-vaccination lobbies? (IL-2)**	Yes	96 (27.1)
	No	258 (72.9)
**In general, do you agree with influential leaders, gatekeepers and anti- or pro-vaccination lobbies? (IL-3)**	Yes	60 (16.9)
	No	294 (83.1)
**WHO Global Vaccine Hesitancy Scale**			
**Have you ever refused a vaccine for yourself or a child because you considered it useless or dangerous?**	Yes	39 (11.0)
	No	315 (89.0)
**Have you ever postponed a vaccine recommended by a physician?**	Yes	39 (11.0)
	No	315 (89.0)
**Have you ever had a vaccine for a child or yourself despite doubts about its efficacy?**	Yes	106 (29.9)
	No	248 (70.1)
**WHO Global Vaccine Hesitancy Scale (Either “Yes” in item VH1 –VH3, if a participant answered yes to one of these statements, he/she was considered to be ‘‘global vaccine-hesitant”)**	Yes	143 (40.4)
	No	211 (59.6)

Note: Data are presented either in Mean ± SD or N (%).

#### Oxford COVID-19 vaccine hesitancy

In terms of vaccine hesitancy, a majority of the participants (88.4%) did not endorse any clear vaccine hesitancy response. More than half of the participants would like to take the COVID-19 vaccine (89.6%), and 312 participants (88.1%) wished to get vaccinated as soon as possible. Participants were found to be keen on getting the COVID-19 vaccine (79.7%) and described themselves as being eager to receive the COVID-19 vaccine (88.4%). They would also encourage others to get the COVID-19 vaccine (85%). Nevertheless, there were 17 participants (4.8%) who responded “probably not” and 12 participants (3.4%) who responded “definitely not” for items 6 and 7 respectively, indicating a clear vaccine hesitancy. The frequencies of endorsement for each of the Oxford COVID-19 Vaccine Hesitancy Scale items are summarized in Appendix Table A1 in S3 File.

#### Pseudoscientific practices

In terms of pseudoscientific practices, 58.8% of the participants reported having used at least one pseudoscientific practice often or very often to prevent a COVID-19 infection. Appendix Table A2 in S3 File shows the frequencies for each pseudoscientific practice used by the participants during the pandemic to prevent a COVID-19 infection, indicating that almost all practices were commonly (with a cumulative percentage of >20%) used (with endorsed responses of “sometimes”, “often”, and “very often”) to prevent themselves from getting the COVID-19 disease, except for item 4 (drank alcoholic beverages item), item 8 (inhaled saline solution), and item 11 (consulted an astrologer). The responses “often” or “very often” (which has a response rating of 4 or 5) were given by participants, particularly on item 1 (drank water every 15 minutes, n = 103 or 29.1%), item 2 (consumed garlic, n = 79 or 22.3%), item 9 (consumed honey or similar products, 23.4%), and item 10 (taken a large amount of Vitamin C, 37%).

#### General vaccination conspiracy beliefs

In terms of general vaccination conspiracy beliefs, a total of 136 participants (38.4%) endorsed at least one of the seven conspiracy belief items, with a response rating of 4 (Agree) or 5 (Strongly agree). A summary of endorsement of each of the General Vaccination Conspiracy Beliefs Scale items is provided in Appendix Table A3 in S3 File.

Notably, it was found that some conspiracy beliefs were commonly accepted (with a cumulative percentage of participants being more than 20% for responses rating of 4 indicating “Agree” or 5 indicating “Strongly agree”). For example, for item 4 –People are deceived about the effectiveness of vaccines, 22.6% of the participants indicated “agree” or “strongly agree with the statement whereas the remaining participants (77.4%) indicated “strongly disagreed” to “neutral” responses. For item 6 –People are deceived about vaccine safety, 22.9% of the participants indicated “agree” or “strongly agree with the statement whereas the remaining participants (77.1%) indicated “strongly disagreed” to “neutral” responses.

#### Subjective norms

The mean score for subjective norms was 22.7 (SD = 5) out of total score of 30. As shown in Appendix Table A4 in S3 File, participants’ acceptance of the COVID-19 vaccine was more likely (endorsed a response rating of “A lot” and “A great deal”) to be influenced by persuasion from doctors (67.2%) as compared to the influence from parents (50%) and a best friend (40.4%).

#### Perceived behavioral control

With regards to perceived behavioural control, around three-fifths (59.3%) of the participants reported a high degree of perceived behavioural control towards vaccination, with a response rating of 4 (agree) or 5 (strongly agree) for all items on the Perceived Behavioral Control Scale. The frequency of each item in perceived behavioural control is shown in Appendix Table A5 in S3 File.

#### Concern about the Halal status of the COVID-19 vaccine

It was noted that slightly more than two-thirds of the participants (72.9%) were not concerned if the COVID-19 vaccine was halal. In the subgroup exploratory analysis (Appendix Table A6 in S3 File), it was noted that slightly more than half of the Malays who are Muslim (51.7%) were concerned if the COVID-19 vaccine was Halal, whereas a majority of non-Malay participants (80.9%) were not concerned about the vaccine’s Halal status.

#### Influential leaders, gatekeepers, and anti-or pro-vaccination lobbies

For the “influential leaders, gatekeepers and anti-or pro-vaccination lobbies” Scale, approximately one-fifth of the participants (19.5%) answered that they either strongly disagreed or disagreed with some groups or leaders who do not agree with vaccination. This study found that a low percentage (27.1%) of participants knew any of the influential leaders, gatekeepers, and anti-or pro-vaccination lobbies and that only 16.9% agree with these groups in general.

#### WHO global vaccine hesitancy

For the WHO Global Vaccine Hesitancy Scale, 143 participants (40.4%) reported that they hesitated to receive the vaccine in general. A total of 39 participants (11.0%) have ever refused a vaccine for themselves or a child because they considered it useless or dangerous; the same number of participants reported that they have ever postponed getting a vaccine recommended by a physician. Also, nearly one-third of the participants (29.9%, n = 106) have ever had a vaccine for a child or themselves despite having doubts about its efficacy.

#### Main reasons for not intending to get the COVID-19 vaccine

Appendix Table A7 in S3 File summarizes the frequency of the 15 main reasons for not intending to get the COVID-19 vaccine. The most highly endorsed reason was being concerned about the side effects and safety of the vaccine (62.1%), followed by not considering themselves to be a member of any group that is at a high risk of contracting the COVID-19 disease (52.8%). Additionally, 45.8% of participants were concerned about the vaccine being developed too quickly. Less than half of the participants endorsed that they planned to use tasks/other precautious instead (41.8%) and planned to wait and see if the vaccine was safe to get and that they may get it at a later time (41.0%).

### Associated factors of COVID-19 vaccine hesitancy

Factors associated with COVID-19 vaccine hesitancy with a p-value of <0.25 using either independent samples t-test or one-way ANOVA (Table 3) and simple linear regression (Table 4) were entered into the multiple variable regression model.

**Table 3:** Factors associated with the Oxford COVID-19 Vaccine Hesitancy Scale using either Independent samples t-test or One-way ANOVA (n = 354).

Factors in categorical data	Category (n value)	Mean (SD)	*p*-values
**Gender**	Male (n = 105)	11.1 (4.6)	0.651
	Female (n = 249)	11.3 (5.0)	
**Ethnicity**	Malay (n = 56)	11.1 (5.6)	0.912
	Chinese (n = 205)	11.2 (4.5)	
	Indian (n = 69)	11.6 (5.5)	
	Others (n = 24)	11.3 (4.1)	
**Education level**	Secondary (n = 24)	12.9 (6.0)	0.243
	Diploma or Bachelor (n = 221)	11.1 (4.5)	
	Postgraduate degree (n = 109)	11.1 (5.3)	
**Household monthly income in Malaysian Ringgit (MYR)**	<MYR 2000 (n = 165)	11.6 (4.7)	0.671
	MYR 2000–3000 (n = 34)	10.6 (5.1)	
	MYR 3000–4000 (n = 19)	10.3 (2.8)	
	MYR 4000–5000 (n = 29)	11.6 (5.4)	
	≥MYR 5000 (n = 107)	11.1 (5.2)	
**Marital status**	Single (n = 247)	11.3 (4.8)	0.366
	Married (n = 9)	11.1 (4.9)	
	Widowed or Divorced (n = 8)	3.6 (6.7)	
**Do you know of any influential, leaders, gatekeepers and anti- or pro-vaccination lobbies? (IL-2)**	No (n = 258)	11.5 (4.6)	0.181
	Yes (n = 96)	10.7 (5.5)	
**In general, do you agree with these groups? (IL-3)**	No (n = 294)	10.8 (4.5)	<0.001
	Yes (n = 60)	13.5 (5.9)	
**WHO Global Vaccine Hesitancy Scale**	Not Hesitant (n = 211)	11.1 (4.7)	0.338
	Hesitant (n = 143)	11.6 (5.1)	

**Table 4:** Factors associated with the Oxford COVID-19 Vaccine Hesitancy Scale using simple linear regression (n = 354).

Associated factors in continuous data	Crude B coefficient (S.E.)	*p*-values
Age	-0.009 (0.019)	0.642
Pseudoscientific practices	0.044 (0.026)	0.088
General conspiracy belief	0.287 (0.035)	<0.001
Subjective norms	-0.497 (0.044)	<0.001
Perceived behavioral control	-0.650 (0.085)	<0.001
Concern if COVID-19 vaccine is Halal	0.197 (0.191)	0.305
Endorsed some groups or leaders who do not agree to vaccination for different reasons (IL-1)	0.375 (0.216)	0.084
The main reason for not intending to get the COVID-19 vaccine		
1. Concern about the side effects and safety of the vaccine	(0.205)	<0.001
2. Concern about the vaccine being developed too quickly	1.280 (0.195)	<0.001
3. Plan to wait and see if it is safe and may get it later	1.652 (0.165)	<0.001
4. Don’t trust the government	1.286 (0.203)	<0.001
5. Plan to use masks/other precautions instead	1.300 (0.185)	<0.001
6. Don’t like vaccines	2.019 (0.209)	<0.001
7. Not a member of any group that is at high risk for COVID-19	0.460 (0.179)	0.011
8. COVID-19 is not a serious illness	1.444 (0.277)	<0.001
9. The vaccine will not work	2.089 (0.214)	<0.001
10. The vaccine will give me COVID-19	1.674 (0.230)	<0.001
11. Had COVID-19 and should be immune	0.369 (0.203)	0.070
12. Don’t like needles	0.618 (0.190)	0.001
13. Doctor has not recommended a COVID-19 vaccine to me	0.815 (0.210)	<0.001
14. Didn’t know I needed a vaccine against COVID-19	1.055 (0.244)	<0.001
15. Concern about the costs associated with the vaccine (such as office visit costs or vaccine administration fees)	0.368 (0.193)	0.057

The results of the multiple linear regression (Table 5) showed that the participants significantly agreed with the following: influential leaders, gatekeepers and anti- or pro-vaccination lobbies (adjusted B coefficient = 1.355, *p* = 0.014), conspiracy beliefs (adjusted B coefficient = 0.128, *p* <0.001), subjective norms (adjusted B coefficient = -0.341, *p* <0.001), perceived behavioral control (adjusted B coefficient = -0.202, *p* = 0.004), planned to wait and see if it is safe and may get it later (adjusted B coefficient = 0.822, *p* <0.001), planned to use masks/others precautions instead (adjusted B coefficient = 0.345, p = 0.038), the vaccine will give them COVID-19 (adjusted B coefficient = 0.660, *p* = 0.002), and concern about the costs associated with the vaccine (such as office visit costs or vaccine administration fees) (adjusted B coefficient = -0.518, *p* = 0.001). The predictors contributed to 53.1% (adjusted R^2^ = 0.531) of the change in the score of COVID-19 vaccine hesitancy, F (23, 330) = 18.368, *p* <0.001.

**Table 5:** Factors associated with Oxford COVID-19 Vaccine Hesitancy Scale with multiple linear regressions (n = 354).

Associated factors	Adjusted B coefficient (S.E)	*p*-values
Highest education level obtained (Secondary school as reference category)	-0.512 (0.335)	0.127
Endorsed some groups or leaders who do not agree to vaccination for different reasons (IL-1)	0.268 (0.155)	0.084
Knew any of these groups or individuals who do not agree to vaccination for different reasons (Did not know as reference category) (IL-2)	-0.207 (0.426)	0.627
In general, I agree with influential leaders, gatekeepers and anti- or pro-vaccination lobbies? (Did not agree as reference category) (IL-3)	1.355 (0.548)	0.014
Pseudoscientific practices	-0.038 (0.023)	0.092
Conspiracy beliefs	0.128 (0.036)	<0.001
Subjective norms	-0.341 (0.041)	<0.001
Perceived behavioral control	-0.202 (0.070)	0.004
The main reason for not intending to get the COVID-19 vaccine		
1. Concern about the side effects and safety of the vaccine	-0.195 (0.202)	0.334
2. Concern that the vaccine is being developed too quickly	0.138 (0.189)	0.457
3. Plan to wait and see if it is safe and may get it later	0.822 (0.172)	<0.001
4. Don’t trust the government	0.053 (0.176)	0.764
5. Plan to use masks/other precautions instead	0.345 (0.165)	0.038
6. Don’t like vaccines	0.328 (0.221)	0.138
7. Not a member of any group that is at high risk for COVID-19	0.056 (0.138)	0.684
8. COVID-19 is not a serious illness	0.081 (0.250)	0.746
9. The vaccine will not work	0.303 (0.236)	0.201
10. The vaccine will give me COVID-19	0.660 (0.216)	0.002
11. Had COVID-19 and should be immune	-0.043 (0.162)	0.790
12. Don’t like needles	-0.201 (0.145)	0.168
13. Doctor has not recommended a COVID-19 vaccine to me	-0.120 (0.169)	0.479
14. Didn’t know I needed a vaccine against COVID-19	0.042 (0.223)	0.849
15. Concern about the costs associated with the vaccine (such as office visit costs or vaccine administration fees)	-0.518 (0.155)	0.001
Model intercept (Constant)	15.965 (1.870)	

Note: Adjusted R^2^ = 0.531, F (23, 330) = 18.368, p<0.001.

## Discussion

### Prevalence of COVID-19 vaccine hesitancy

In this study, we observed that 11.6% of the participants were hesitant to receive the COVID-19 vaccine based on the assessment done using the Oxford COVID-19 Vaccine Hesitancy Scale. However, when it came to non-specific vaccines, 40.4% of respondents were hesitant to get these vaccines, as seen in the assessment done using the WHO Global Vaccine Hesitancy Scale. The difference in findings between these two scales indicated that respondents were more accepting of the COVID-19 vaccine than any other type of vaccine for other diseases. This could be due to the propaganda and scientific facts surrounding the benefits of receiving the COVID-19 vaccines that generally outweigh the harm to mankind, which can be widely seen circulating on social media and the community at large. The publicity and intensive discussion people have on the COVID-19 vaccine as compared to any other kinds of vaccines for other diseases is unprecedented. Therefore, the percentage of participants who were hesitant about getting the COVID-19 vaccine was much lower than for other non-specific vaccines. On top of that, with the government imposing mandatory COVID-19 vaccination orders on those entering malls and stores, the mobility of unvaccinated people will be restricted. This could be another factor "forcing" people to accept the COVID-19 vaccines. The review by Sallam et al. [24] reported that the COVID-19 vaccine hesitancy or rejection rate in Asia is highest in Hong Kong (60%), and the lowest in Indonesia (6.7%). Our findings on COVID-19 vaccine hesitancy displayed a higher percentage than the reported result of 5.7% by Wong et al., 2020 [8]. One of the possible factors for the difference could be due to the demographic makeup of the participants in this study, where nearly half of the participants (46.6%) in this study were from the lower strata income group (<MYR 2000 per month), which constituted a proportion that is almost four times higher as compared to the percentage of participants (12.3%) from the lower strata income group in the study by Wong et al., [8]. Financial status was associated with the level of education achieved. It was found that the majority of participants with monthly household incomes of less than MYR 2000 were those with a secondary level of education (79.2%) as compared to 16.5% of participants who held a postgraduate degree and 57.9% of participants who had a diploma and graduate degrees.

A study by Mohamed et al. [30] conducted among Malaysians showed that a higher education level was significantly associated with a higher level of vaccine acceptance. On the other hand, those who come from a low educational background and are of lower socioeconomic status may be lacking in knowledge of the importance of vaccination, which may, in turn, influence their intention to get the vaccine. Our explanation for the difference in vaccine hesitancy between that of Wong et al. [8] and this study is consistent with previous studies which stated that financial status affects the attitude toward COVID-19 vaccination [31–33].

### Associated factors for COVID-19 vaccine hesitancy

We obtained several significant findings from the expanded TPB Model. We found that COVID-19 vaccine hesitancy was significantly positively associated with those who agreed with influential leaders, gatekeepers, and anti- or pro-vaccination lobbies, those who had a higher score in conspiracy beliefs, those who would wait and see if the COVID-19 vaccine was safe and may then opt to get it later, and those who perceived that the vaccine would give them COVID-19. Conversely, subjective norms perceived behavioural control, and concern about the costs associated with the vaccine was negatively associated with COVID-19 vaccine hesitancy.

Our results showed that participants who agreed with influential leaders, gatekeepers and anti- or pro-vaccination lobbies were more hesitant toward getting the COVID-19 vaccine. Perhaps our participants had received misguided anti-vaccination messages or were exposed to non-authoritative social media coverages that influenced their attitudes toward vaccines despite the pro-vaccination campaigns delivered by health professionals through official channels.

Many perceived cultural or religious convictions as vaccination barriers have been reported in previous studies [34,35]. This may be associated with the multi-ethnic and multi-religious nature of the participants in relation to the demographic makeup of Malaysia. This could be true, as previous studies reported that religious influencers have been shown to have a contingent influence on people’s attitudes during a crisis [36,37]. Therefore, if religious influencers used religious and moral convictions in their anti-vaccination messages, their speech could have indirectly discouraged people from getting vaccinated. Of note, there were false claims that vaccine ingredients contained cells of aborted fetuses, animal genes, toxic ingredients, microchips, DNA modifiers, or multipliers that could permanently alter our DNA [34,35]. The low level of trust in the safety and efficacy of the COVID-19 vaccine may also arise from the perceived low representation of local communities in vaccine trials and that they (nationals from countries that did not invent the vaccine) were treated as an experimental unit for the vaccine [35].

Our findings showed that the endorsement of conspiracy beliefs was associated with COVID-19 vaccine hesitancy. The characteristics of the seven items of general vaccination conspiracy beliefs used in this study had largely dealt with the fabrication of facts about safety and efficacy aspects. It is not surprising to see this result, as multiple studies have reported similar findings [38,39]. In addition, another study found that conspiracy theories with either political or economic motives (e.g., the vaccination program was construed as controlling humanity with artificial intelligence or introducing the “World Debt Reset Program” or “New World Order”) contributed to vaccine hesitancy [35]. Taking a closer look into the descriptive analysis on each item of the scale, it was shown that item 4 (People are deceived about the effectiveness of vaccines) and item 6 (People are deceived about vaccine safety) were perceived by more than 20% of participants (with a cumulative percentage of participants responding “Agree” or “Strongly agree”). This could be due to the persuasion of the government towards the public that COVID-19 vaccines are safe and effective in spite of the current news that point to evidences showing how the COVID-19 vaccine has led to higher rates of injury and death in certain populations, of which was not acknowledged by the health authorities and made transparent in reports to the public.

Pseudoscientific practices were found to not be associated with COVID-19 vaccine hesitancy; however, there are some findings from the descriptive analysis that is worth mentioning. For instance, it was found that the majority of pseudoscientific practices (except for item 4 –drinking alcoholic beverages; item 8 –inhaled saline solution; item 11 –consulted an astrologer) were common (with a cumulative percentage of >20% for the responses “sometimes”, “often: and “very often”). A possible explanation behind the beliefs people hold towards pseudoscientific practices in preventing COVID-19 is that people were more frequently exposed to and influenced by varying statements, claims, and pseudoscientific language and jargon surrounding preventive measures against COVID-19, all of which give the false impression that these measures have been shown, via laboratory testing and research, to be legitimate and effective.

We found that agreements to the statements “wait and see if COVID-19 vaccine is safe and may then get it later”, “planned to use masks/other precautions instead”, and “perceived that the vaccine against COVID-19 will give them COVID-19” imply vaccine hesitancy. The “wait and see” attitude reflects a lack of confidence in the safety of the vaccine. Multiple qualitative studies and online surveys reported concerns regarding vaccine side effects and fear/worry about the unforeseen future effects of the vaccine, especially on children, the elderly, individuals with comorbid disorders, and immunocompromised patients [40–42]. To overcome the worries, the public should be educated with facts regarding the low prevalence of mortality following COVID-19 vaccination, which only occurs at the rate of one per million in a vaccinated population. They should also be reassured that one’s health conditions will be evaluated carefully, especially among those with underlying diseases, to ensure that they are eligible to receive the vaccine that is compatible with their underlying health conditions. Even though governments have already disseminated such information, this information might not reach everyone, partly due to language barriers, or language preference when sourcing more information before making a decision [41]. Therefore, a wide range of commonly used languages should be considered in the campaigns promoting vaccine acceptance. In addition, scepticism concerning the COVID-19 vaccine may soon diminish as more vaccinated people do not exhibit serious side effects, such as those who safely received the Oxford-AstraZeneca vaccine locally, even though some cases of blood clotting incidences were reported overseas [43].

“Planned to use masks/other precautions instead” was found to be one of the predictors of COVID-19 vaccine hesitancy. Currently, the government has made it mandatory for people to wear face masks in public areas to protect themselves and others around them from getting an infection. Additionally, regular disinfection of surfaces, applying hand sanitisers, and wearing face protectors are some other safety measures that are widely accepted and commonly practised by the public. With regards to the effectiveness of these measures in preventing the spread of the COVID-19 virus, a review, for instance, reported that wearing a face mask was associated with a significantly reduced risk of a COVID-19 infection (pooled odd ratio = 0.38; 95% confidence interval: 0.21–0.69) [44]. When it comes to vaccination, despite the odds being significantly lower for those vaccinated becoming severely ill and subsequently in need of hospitalization as compared to their unvaccinated counterparts, the COVID-19 vaccines are not expected to render all those who receive it impervious to infection. The scientific community has also been unable to satisfactorily show that the COVID-19 vaccine is perfectly safe to get and completely effective in preventing the spread of COVID-19. Such inconsistencies perhaps bring about confusion for many and become a factor in vaccine hesitancy. On top of that, unfavourable testimonies and misleading information about the safety of COVID-19 vaccines give people a false sense of insecurity [45]. Thus, among some people, the wearing of face masks and practices of preventive measures as precautions are taken instead, which are perceived as physical barriers that aid in preventing the transmission of the virus from one person to another.

The endorsement of statements such as “Perceived that the vaccine against COVID-19 will give them COVID-19” reveals that some of the study participants were misinformed about the vaccine. The misinformation could have been disseminated on social media or telecommunication sites [46]. One of the possible hypotheses is that people know that vaccines contain weakened or inactive parts of the COVID-19 virus as baits to trigger an immune response, therefore they feared that the vaccine will give them the virus. A qualitative study reported that individuals felt overwhelmed and confused by the wide variety of sources of information about COVID-19, such as the television, radio, YouTube, Facebook, and council websites. Participants agreed that some of the information was too distressing and did not make sense [41,45]. We should bear in mind that those with low health literacy and lower educational attainment may react differently to the misinformation [34]. Therefore, curbing the rise of misinformation, educating the public on how to seek reliable sources of information, and identifying false or inaccurate information are essential tasks that need to be considered seriously in this information era to prevent a crisis of trust in the authorities and health services.

Additionally, we found that subjective norms, perceived behavioural control, and concern about the costs associated with the vaccine was negatively associated with COVID-19 vaccine hesitancy. First, a high score in subjective norms implies that the study participants tended to be influenced by others (parents, doctors, and best friends) favouring vaccination behaviour and having a stronger intention to get vaccinated. In this case, the positive influence of social norms outweighed the barriers.

Second, a high score in perceived behavioural control emerged as one of the significant predictors that negatively influenced vaccine hesitancy. Perhaps those with a high level of perceived behavioural control have adequate education and the ability to overcome the barriers to getting vaccinated [47]. However, we did not find education level and monthly household income to be associated with the score of perceived behavioural control. Upon further examination, we found that perceived behavioural control was inversely associated with endorsement of the majority of the main reasons for not intending to get the COVID-19 vaccine, except for “concern about the side effect and safety of the vaccine”, “had COVID-19 and should be immune”, “don’t like needles”, “didn’t know I needed a vaccine against COVID-19”, and “concern about the costs associated with the vaccine”. Based on the findings, it is implied that those with a high score in perceived behavioural control are those who can overcome misinformation, doubts, or myths, as well as negative perceptions circulating among the minority of anti-vaxxers and fence-sitters. Perhaps they used scientific facts to overcome these barriers. On the other hand, those with a low score in perceived behavioural control may be easily shaken in their trust or confidence in the COVID-19 vaccine when exposed to anti-vaccination sentiments.

Third, “concern about the costs associated with the vaccine” was negatively associated with vaccine hesitancy. This could be explained by the fact that individuals who are willing to pay for the vaccine may perceive the vaccine to be worth their financial investment, and therefore may be less hesitant to take the vaccine.

This study had some limitations. Since the demographic characteristics, particularly on ethnicity distribution, did not reflect the actual demographics of Malaysia, there is limited generalizability of the findings. Second, this study was limited to using online self-report measures of vaccine hesitancy rather than using an objective measure and may thus be subjected to social desirability bias. Third, we did not obtain the official religion of the participants. Even though most Malays in Malaysia are Muslim, there may be non-Malays who are also Muslims. This assumption is supported by the fact that around one-tenth of non-Malays were very concerned about whether the vaccine was Halal. Therefore, we were not able to determine the Muslim and non-Muslims’ perspectives on the Halal issue surrounding the COVID-19 vaccine. Fourth, this study has some degree of sampling bias as an exponential non-discriminative snowballing sampling method was used to recruit participants, which might not be representing the targeted population or resembling the demographic of Malaysia. Fifth, the three items regarding the “influential leaders, gatekeepers and anti-or pro-vaccination lobbies” Scale (Section 8) could be interpreted ambiguously, whereby the wording “anti” and “pro” vaccination may have confused our participants Therefore, the results for this section must be interpreted cautiously.

## Conclusions

In this study, we found three significant determinants of COVID-19 vaccine hesitancy, which are a) influenced by leaders, and anti- or pro-vaccination lobbies, b) belief in conspiracy theories, and c) lack of trust in the vaccine’s safety. On the other hand, lower scores on subjective norms and perceived behavioural control, and being less concerned about the costs associated with the COVID-19 vaccine were predictors against COVID-19 vaccine hesitancy. Overall, this study indicated that the variables (i.e., subjective norms used to represent normative belief and perceived behavioural control used to represent control belief) in the context of the Theory of Planned Behavior model were partly appropriate in explaining COVID-19 vaccine hesitancy; on the other hand, behavioural belief (which was represented by WHO Global COVID-19 vaccine hesitancy) did not explain COVID-19 vaccine hesitancy. On another note, the expanded TPB model (with the inclusion of pseudoscientific practices, general vaccination conspiracy beliefs, and main reasons for not intending to get the COVID-19 vaccine), was proven to be particularly relevant in articulating the predictors of COVID-19 vaccine hesitancy. Based on these findings, we conclude that the expanded TPB model should be utilized by public health policymakers in constructing.

## Supporting information

S1 File Excel file for data set. (XLSX)

S2 File (PDF)

S3 File (DOCX)
