# Supplementary material for: COVID-19 vaccine hesitancy and its associated factors in Malaysia
Source: PLoS One. 2022 Sep 1;17(9):e0266925. doi: 10.1371/journal.pone.0266925 (PMC9436036; doi:10.1371/journal.pone.0266925)
Supplement: S2 File — (PDF) [file pone.0266925.s002.pdf]

COVID-19 Vaccine Hesitancy and its Associated Factors in Malaysia Survey

Section 1: Demographics

No	Question	Choices
1	Age	State
2	Gender	☐ Female<br>☐ Male
3	Ethnicity	☐ Malay<br>☐ Chinese<br>☐ Indian<br>☐ Others
4	Highest education level	☐ Primary<br>☐ Secondary<br>☐ Diploma and Degree<br>☐ Postgraduate (Masters and PhD)
5	Income level	☐ Less than RM 2000<br>☐ RM 2000 to RM 3000<br>☐ RM 3000 to RM 4000<br>☐ RM 4000 to RM 5000<br>☐ More than RM 5000
6	Family status	☐ Single<br>☐ Married<br>☐ Divorced<br>☐ Widowed
7	Location	☐ Kedah<br>☐ Perlis<br>☐ Penang<br>☐ Terengganu<br>☐ Perak<br>☐ Selangor<br>☐ Kelantan<br>☐ Pahang<br>☐ Negeri Sembilan<br>☐ Melaka<br>☐ Johor<br>☐ Sabah<br>☐ Sarawak<br>☐ Kuala Lumpur<br>☐ Putrajaya<br>☐ Labuan
8	I am concern if the new COVID-19 vaccine is halal.	☐ Strongly concern<br>☐ Not concerned<br>☐ Neutral<br>☐ Concerned<br>☐ Strongly concerned

Section 2: Vaccination hesitancy and intention

We are interested in your perception of getting the COVID-19 vaccination. Using the 5-point scale shown below, Read the statement below and select your response based on your current situation.

Select Don't know if you are unsure or do not know.

1. Would you take a COVID-19 vaccine (approved for use in the Malaysia) if offered?

Definitely<br>1	Probably<br>2	I may or may not<br>3	Probably not<br>4	Definitely not<br>5	Don't know

2. If there was a Covid-19 vaccine available:

I will want to get it as soon as possible<br>1	I will take it when offered<br>2	I'm not sure what I will do<br>3	I will put off (delay) getting it<br>4	I will refuse to get it<br>5	Don't know

3. I would describe my attitude towards receiving a Covid-19 vaccine as:

Very keen<br>1	Pretty positive<br>2	Neutral<br>3	Quite uneasy<br>4	Against it<br>5	Don't know

4. I would describe myself as:

Eager to get a Covid-19 vaccine<br>1	Willing to get the Covid-19 vaccine<br>2	Not bothered about getting the Covid-19 vaccine<br>3	Unwilling to get the Covid-19 vaccine<br>4	Anti-vaccination for Covid-19<br>5	Don't know

5. If my family or friends were thinking of getting a COVID-19 vaccination, I would:

Strongly encourage them<br>1	Encourage them<br>2	Not say anything to them about it<br>3	Ask them to delay getting the vaccination<br>4	Suggest that they do not get the vaccination<br>5	Don't know

6. If a COVID-19 vaccine was available at my local pharmacy, I would:

**Get it as soon as possible 1**	**Get it when I have time 2**	**Delay getting it 3**	**Avoid getting it for as long as possible 4**	**Never get it 5**	**Don't know**

7. Taking a COVID-19 vaccination is:

**Really important 1**	**Important 2**	**Neither important nor unimportant 3**	**Unimportant 4**	**Really unimportant 5**	**Don't know**

Section 3: Pseudoscientific belief

In the last 2 weeks, I have engaged in the following behaviors with the aim of preventing to contract coronavirus.

**1**	**2**	**3**	**4**	**5**
**Never**	**Rarely**	**Sometimes**	**Often**	**Very often**

1	Drank water every 15 min	
2	Consumed garlic	
3	Using colloidal silver	
4	Drank alcoholic beverages	
5	Drank ginger tea, baking soda with lemon or similar drinks	
6	Used essential oils	
7	Followed a special diet	
8	Inhaled saline solution	
9	Consumed honey or similar products	
10	Taken large amounts of vitamin C	
11	Consulted an astrologer	
12	Disinfected surfaces with natural products (eg. Vinegar, baking soda)	

Section 4: General vaccinate conspiracy belief

Using the 5-point scale shown below, please state your level of agreement with the statement below.

1	2	3	4	5
**Strongly disagree**	**Disagree**	**Neutral**	**Agree**	**Strongly agree**

1	Vaccine safety data are often fabricated (made up).	
2	Immunising children is harmful and this fact is covered up.	
3	Pharmaceutical companies cover up the dangers of vaccines.	
4	People are deceived about the effectiveness of vaccines	
5	Vaccine effectiveness data are often fabricated (made up).	
6	People are deceived about vaccine safety.	
7	The government is trying to cover up the link between vaccines and autism.	

Section 5: Subjective Norms

Using the 5-point scale shown below, please state your level of agreement with the statement below.

1	2	3	4	5
**Not at all**	**A little**	**Somewhat**	**A lot**	**A great deal**

1	How much do your parents think you should receive the Covid-19 vaccine?	
2	How much does your doctor think you should receive the Covid-19 vaccine?	
3	How much does your best friend think you should receive the Covid-19 vaccine?	
4	In general, I want to do what my parents think I should do.	
5	In general, I want to do what my doctor thinks I should do.	
6	In general, I want to do what my best friend thinks I should do	

Section 6: Perceived Behavioural Control (PBC)

Using the 5-point scale shown below, please state your level of agreement with the statement below.

1	2	3	4	5
**Strongly disagree**	**Disagree**	**Neutral**	**Agree**	**Strongly agree**

1	If I wanted to, I could attend an appointment to get vaccinated against Covid-19.	
2	I feel confident in my ability to get vaccinated for Covid-19.	
3	There are (no) barriers in the way of me receiving the Covid-19 vaccine.	

Section 7: Influential leaders, gatekeepers and anti- or pro-vaccination lobbies

Using the 5-point scale shown below, please state your level of agreement with the statement # 1 below.

1	2	3	4	5
**Strongly disagree**	**Disagree**	**Neutral**	**Agree**	**Strongly agree**

1	Some groups or leaders do not agree to vaccination for different reasons	

**For below two questions, please indicate by using Yes or No**		
2	Do you know of any of these groups or individuals?	**Yes/No**
3	In general, do you agree with these groups?	**Yes/No**

Section 8: The Vaccine hesitancy

**For below three question, please indicate by using Yes or No**			
1	Have you ever refused a vaccine for yourself or a child because you considered it as useless or dangerous?	**Yes**	**No**
2	Have you ever postponed a vaccine recommended by a physician?	**Yes**	**No**
3	Have you ever had a vaccine for a child or yourself despite doubts about its efficacy?	**Yes**	**No**

Section 9: Reasons for vaccine hesitancy

Using the 5-point scale shown below, please state your level of agreement with the statements below.

1	2	3	4	5
**Strongly disagree**	**Disagree**	**Neutral**	**Agree**	**Strongly agree**

1	I am concern about the side effects and safety of the vaccine	
2	I am concern that the vaccine is being developed too quickly	
3	I plan to wait and see if it is safe and may get it later	
4	I don’t trust the government	
5	I plan to use masks/other precautions instead	
6	I don’t like vaccines	
7	I am not a member of any group that is at high risk for COVID-19	
8	COVID-19 is not a serious illness	
9	The vaccine will not work	
10	The vaccine could give me COVID-19	
11	Had COVID-19 and should be immune	
12	I don’t like needles	
13	Doctor has not recommended a COVID-19 vaccine to me	
14	I don’t know I needed a vaccine against COVID-19	
15	I am concern about the costs associated with the vaccine (such as office visit costs or vaccine administration fees)	
