# Supplementary material for: COVID-19 vaccine hesitancy and its associated factors in Malaysia
Source: PLoS One. 2022 Sep 1;17(9):e0266925. doi: 10.1371/journal.pone.0266925 (PMC9436036; doi:10.1371/journal.pone.0266925)
Supplement: S3 File — (DOCX) [file pone.0266925.s003.docx]

| Table A1. Descriptive analysis of the endorsement of Oxford COVID-19 Vaccine Hesitancy Scale – 7 items (n=354). | | | | | | |
| --- | --- | --- | --- | --- | --- | --- |
|  | **Strongly Disagree**  (scored as 1) | **Disagre**e (scored as 2) | **Neutral** (scored as 3) | **Agree** (scored as 4) | **Strongly Agree** (scored as 5) | **Don’t know**  scored as 0) |
| 1. Would you take a COVID-19 vaccine (approved for use in Malaysia) if offered?   (1=Definitely to 5=Definitely not) | 254  (74.9) | 52  (14.7) | 24  (6.8) | 8  (2.3) | 3  (0.8) | 2  (0.6) |
| 1. If there was a COVID -19 vaccine available   (1=I will want to get it as soon as possible to 5=I will refuse to get it, 0=don't know) | 197  (55.6) | 115  (32.5) | 14  (4.0) | 19  (5.4) | 6  (1.7) | 3  (0.8) |
| 1. I would describe my attitude towards receiving a COVID -19 vaccine as:   (1=Very keen to 5=against it) | 143  (40.4) | 139  (39.3) | 48  (13.6) | 18  (5.1) | 4  (1.1) | 2  (0.6) |
| 1. I would describe myself as:   (1=Eager to get a COVID -19 vaccine to 5=anti-vaccination for COVID -19) | 132  (37.3) | 181  (51.1) | 20  (5.6) | 12  (3.4) | 5  (1.4) | 4  (1.1) |
| 1. If my family or friends were thinking of getting a COVID-19 vaccination, I would:   (1=Strongly encourage them to 5=suggest that they do not get the vaccination) | 160  (45.2) | 141  (39.8) | 44  (12.4) | 5  (1.4) | 2  (0.6) | 2  (0.6) |
| 1. If a COVID-19 vaccine was available at my local pharmacy, I would:   (1=Get it as soon as possible to 5=never get it) | 224  (63.3) | 85  (24.0) | 20  (5.6) | 13  (3.7) | 4  (1.1) | 8  (2.3) |
| 1. Taking a COVID-19 vaccination is:   (1=Really important to 5= really unimportant) | 243  (68.6) | 77  (21.8) | 19  (5.4) | 6  (1.7) | 6  (1.7) | 3  (0.8) |
| Note: Data are presented in n (percentage). | | | | | | |

| Table A2. Descriptive analysis of Pseudoscientific Practices Scale during the COVID-19 pandemic – 12 items (n=354). | | | | | |
| --- | --- | --- | --- | --- | --- |
| In the last 2 weeks, how often have you engaged in the following practices with the aim of preventing COVID-19 | **Never**  (scored as 1) | **Rarely**  (scored as 2 | **Sometimes**  (scored as 3) | **Often**  (scored as 4) | **Very often** (scored as 5) |
| 1. Drank water every 15 minutes | 107 (30.2) | 56 (15.8) | 88 (24.9) | 64 (18.1) | 39 (11.0) |
| 1. Consumed garlic | 132 (37.3) | 73 (20.6) | 70 (19.8) | 43 (12.1) | 36 (10.2) |
| 1. Using colloidal silver | 200 (56.5) | 66 (18.6) | 75 (21.2) | 9 (2.5) | 4 (1.1) |
| 1. Drank alcoholic beverages | 250 (70.6) | 44 (12.4) | 36 (10.2) | 20 (5.6) | 4 (1.1) |
| 1. Drank ginger tea, baking soda with lemon or similar drinks | 173 (48.9) | 53 (15.0) | 69 (19.5) | 34 (9.6) | 25 (7.1) |
| 1. Used essential oil | 187 (52.8) | 61 (17.2 | 61 (17.2) | 31 (8.8) | 14 (4.4) |
| 1. Followed a special diet | 193 (54.5) | 51 (14.4) | 60 (16.9) | 38 (10.7) | 12 (3.4) |
| 1. Inhaled saline solution | 229 (64.7) | 64 (18.1) | 46 (13.0) | 9 (2.5) | 6 (1.7) |
| 1. Consumed honey or similar products | 159 (44.9) | 49 (13.8) | 63 (17.8) | 60 (16.9) | 23 (6.5) |
| 1. Taken large amount of Vitamin C | 93 (26.3) | 52 (14.7) | 78 (22.0) | 79 (22.3) | 52 (14.7) |
| 1. Consulted an astrologer | 253 (71.5) | 42 (11.9) | 47 (11.3) | 7 (2.0) | 5 (1.4) |
| 1. Disinfected surfaces with natural (e.g., vinegar or baking soda) | 198 (55.9) | 44 (12.4) | 67 (18.9) | 24 (6.8) | 21 (5.9) |
| Note: Data are presented in n (percentage). | | | | | |

| Table A3. Endorsement of General Vaccination Conspiracy Beliefs Scale– 7 items (n=354). | | | | | |
| --- | --- | --- | --- | --- | --- |
| General Vaccination Conspiracy Belief | **Strongly disagree** (scored as 1) | **Disagree**  (scored as 2) | **Neutral**  (scored as 3) | **Agree**  (scored as 4) | **Strongly agree**  (scored as 5) |
| 1. Vaccine safety data are often fabricated (made up) | 124 (35.0) | 64 (18.1) | 110 (31.1) | 39 (11.0) | 17 (4.8) |
| 1. Immunizing children is harmful, and this fact is covered up | 158 (44.6) | 64 (18.1) | 91 (25.7) | 27 (7.6) | 14 (4.0) |
| 1. Pharmaceutical companies cover up the dangers of vaccines | 96 (27.1) | 73 (20.6) | 120 (33.9) | 42 (11.9) | 23 (6.5) |
| 1. People are deceived about the effectiveness of vaccines | 97 (27.4) | 64 (18.1) | 113 (31.9) | 49 (13.8) | 31 (8.8) |
| 1. Vaccine effectiveness data are often fabricated (made up) | 107 (30.2) | 93 (26.3) | 106 (29.9) | 33 (9.3) | 15 (4.2) |
| 1. People are deceived about vaccine safety | 89 (25.1) | 82 (23.2) | 102 (28.8) | 50 (14.1) | 31 (8.8) |
| 1. The government is trying to cover up the link between vaccines and autism | 147 (41.5) | 63 (17.8) | 105 (29.7) | 23 (6.5) | 16 (4.5) |
| Note: Data are presented in n (percentage). | | | | | |

| Table A4. Descriptive analysis of Subjective Norms Scale – 6 items (n=354). | | | | | |
| --- | --- | --- | --- | --- | --- |
| Subjective norms | **Not at all**  (Scored as 1) | **A little**  (Scored as 2) | **Somewhat**  (Scored as 3) | **A lot**  (Scored as 4) | **A great deal**  (Scored as 5) |
| 1. How much do your parents think you should receive the COVID-19 vaccine | 19 (5.4) | 18 (5.1) | 76 (21.5) | 94 (21.5) | 147 (41.5) |
| 1. How much does your doctor think you should receive the COVID-19 vaccine | 4 (1.1) | 4 (1.1) | 79 (22.3) | 84 (23.7) | 183 (51.7) |
| 1. How much does your best friend think you should receive the COVID-19 vaccine | 4 (1.1) | 11 (3.1) | 82 (23.2) | 102 (28.8) | 155 (43.8) |
| 1. In general, I want to do what my parents think I should do | 48 (13.6) | 38 (10.7) | 91 (25.7) | 85 (24.0) | 92 (26.0) |
| 1. In general, I want to do what my doctor thinks I should do | 20 (5.6) | 14 (4.0) | 82 (23.2) | 120 (33.9) | 118 (33.3) |
| 1. In general, I want to do what my best friend think I should do | 53 (15.0) | 43 (12.1) | 115 (32.5) | 62 (17.5) | 81 (22.9) |
| Note: Data are presented in n (percentage). | | | | | |

| Table A5. Descriptive analysis of Perceived Behavior Control Scale– 3 items (n=354). | | | | | |
| --- | --- | --- | --- | --- | --- |
|  | **Strongly disagree** (scored as 1) | **Disagree**  (scored as 2) | **Neutral**  (scored as 3) | **Agree**  (scored as 4) | **Strongly agree**  (scored as 5) |
| 1. If I wanted to, I could attend an appointment to get vaccinated against COVID-19 | 23 (6.5) | 19 (5.4) | 44 (12.4) | 116 (32.8) | 152 (42.9) |
| 1. I feel confident in my ability to get vaccinated for COVID-19 | 6 (1.7) | 18 (5.1) | 52 (14.7) | 136 (38.4) | 142 (40.1) |
| 1. There are (no) barriers in the way of me receiving the COVID-19 vaccine | 19 (5.4) | 39 (11.0) | 62 (17.5) | 111 (31.4) | 123 (34.7) |
| Note: Data are presented in n (percentage). | | | | | |

**Table A6. Subgroup analysis between ethnicity for concern over whether the COVID-19 vaccine is Halal (n=354).**

| Concern about whether the COVID-19 vaccine is Halal | Strongly not concerned  (scored as 1) | Not concerned  (scored as 2) | Neutral  (scored as 3) | Concerned  (scored as 4) | Strongly concerned  (scored as 5) |
| --- | --- | --- | --- | --- | --- |
| Malay | 14 (25.0) | 3 (5.4) | 10 (17.9) | 11 (19.6) | 18 (32.1) |
| Non-Malay | 221 (74.2) | 20 (6.7) | 34 (11.4) | 7 (2.3) | 16 (5.4) |
| Note: Data are presented in n (percentage). | | | | | |

| Table A7. Descriptive analysis of main reasons for not intending to get the COVID-19 vaccine – 15 items (n=354). | | | | | |
| --- | --- | --- | --- | --- | --- |
| Main reasons for not intending to get the COVID-19 vaccine | **Strongly disagree** (Scored as 1) | **Disagree**  (scoredas 2) | **Neutral**  (scoredas 3) | **Agree**  (scoredas 4) | **Strongly agree**  (scored as 5) |
| 1. Concern about the side effects and safety of the vaccine | 26 (7.3) | 41 (11.6) | 67 (18.9) | 120 (33.9) | 100 (28.2) |
| 1. Concern about the vaccine is being developed too quickly | 41 (11.6) | 60 (16.9) | 91 (25.7) | 98 (27.7) | 64 (18.1) |
| 1. Plan to wait and see if it is safe and may get it later | 66 (18.6) | 58 (16.4) | 85 (24.0) | 71 (20.1) | 74 (20.9) |
| 1. Don’t trust the government | 49 (13.8) | 80 (22.6) | 114 (32.2) | 66 (18.6) | 45 (12.7) |
| 1. Plan to use masks/other precautions instead | 52 (14.7) | 47 (13.3) | 107 (30.2) | 73 (20.6) | 75 (21.2) |
| 1. Don’t like vaccines | 164 (46.3) | 89 (25.1) | 69 (19.5) | 18 (5.1) | 14 (4.0) |
| 1. Not a member of any group that is at high risk for COVID-19 | 53 (15.0) | 47 (13.3) | 67 (18.9) | 72 (20.3) | 115 (32.5) |
| 1. COVID-19 is not a serious illness | 262 (74.0) | 48 (13.6) | 25 (7.1) | 13 (3.7) | 6 (1.7) |
| 1. The vaccine will not work | 158 (44.6) | 84 (23.7) | 80 (22.6) | 23 (6.5) | 9 (2.5) |
| 1. The vaccine will give me COVID-19 | 171 (48.3) | 76 (21.5) | 81 (22.9) | 18 (5.1) | 8 (2.3) |
| 1. Had COVID-19 and should be immune | 148 (41.8) | 59 (16.7) | 86 (24.3) | 38 (10.7) | 23 (6.5) |
| 1. Don’t like needles | 126 (35.6) | 57 (16.1) | 85 (24.0) | 53 (15.0) | 33 (9.3) |
| 1. Doctor has not recommended a COVID-19 vaccine to me | 138 (39.0) | 79 (22.3) | 91 (25.7) | 22 (6.2) | 24 (6.8) |
| 1. Didn’t know I needed a vaccine against COVID-19 | 219 (61.9) | 64 (18.1) | 49 (13.8) | 10 (2.8) | 12 (3.4) |
| 1. Concern about the costs associated with vaccine (such as office visit costs or vaccine administration fees | 143 (40.3) | 58 (16.4) | 72 (20.3) | 54 (15.3) | 27 (7.6) |
| Note: Data are presented in n (percentage). | | | | | |
